# Fast Coding Unit Encoding Mechanism for Low Complexity Video Coding

**DOI:** 10.1371/journal.pone.0151689

**Published:** 2016-03-21

**Authors:** Yuan Gao, Pengyu Liu, Yueying Wu, Kebin Jia, Guandong Gao

**Affiliations:** 1 Beijing Advanced Innovation Center for Future Internet Technology, Beijing University of Technology, Beijing, China; 2 Beijing Laboratory of Advanced Information Networks, Beijing, China; 3 College of Electronic Information and Control Engineering, Beijing University of Technology, Beijing, China; Huazhong University of Science and Technology, CHINA

## Abstract

In high efficiency video coding (HEVC), coding tree contributes to excellent compression performance. However, coding tree brings extremely high computational complexity. Innovative works for improving coding tree to further reduce encoding time are stated in this paper. A novel low complexity coding tree mechanism is proposed for HEVC fast coding unit (CU) encoding. Firstly, this paper makes an in-depth study of the relationship among CU distribution, quantization parameter (QP) and content change (CC). Secondly, a CU coding tree probability model is proposed for modeling and predicting CU distribution. Eventually, a CU coding tree probability update is proposed, aiming to address probabilistic model distortion problems caused by CC. Experimental results show that the proposed low complexity CU coding tree mechanism significantly reduces encoding time by 27% for lossy coding and 42% for visually lossless coding and lossless coding. The proposed low complexity CU coding tree mechanism devotes to improving coding performance under various application conditions.

## 1. Introduction

A new birth after a decade of preparations, HEVC [1] has been initiated by the Joint Collaborative Team on Video Coding (JCT-VC), jointly established by the International Organization for Standardization (ISO)/ International Electrotechnical Commission (IEC) Moving Picture Experts Group (MPEG) and International Telecommunication Union–Telecommunication (ITU-T) Video Coding Experts Group (VCEG). Due to the rapid growth of multimedia services, realizing HEVC in applications while simultaneously providing high quality images with minimum transmission delay over the limited rate networks has remained a challenge [2–3]. HEVC is expected to have more impact on high resolution video (4K and 8K) or high fidelity video for high resolution displays such as high definition TV (HD-TV) and ultrahigh definition TV (UHD-TV) [4–5] in the future. HEVC not only inherits the crucial elements of H.264/AVC, but also accepts numerous new techniques [6–7] to achieve considerable performance. Among these advanced techniques, coding tree structure is employed as one of the most powerful tools to improve the coding efficiency of HEVC. However coding tree is a complicated technique that contributes to improving compression performance at the cost of increasing huge computational complexity. Computational complexity reduction with negligible performance loss has been one of the major research concerns, because videos may be played in any kind of device such as a PC or mobile phone with different computational abilities and system resources.

Recently, a large amount of effort has been made for CU coding tree computational complexity reduction for HEVC. Studies on fast CU encoding improvement tried to represent the redundant information that often exists in CU coding tree traversal computing. Traditionally, dynamic information redundancy in videos occurs in the spatial and temporal sense. Thus, alternative mechanisms for intra mode decision and inter mode decision would be interesting. Many works have focused on the development of fast coding tree computing by early CU size decision for HEVC intra encoders [8–10] and inter encoders [11–12]. Shen [8] skips intra prediction modes that are rarely used in the parent CUs in the upper depth levels or spatially nearby CUs. Ahn [12] simplified the RD competition processes by selectively conducting a mode decision process according to inter predicting unit split-type, square-type, or non-square-type modes. These approaches effectively reduced encoding time by mostly using fast mode decision in predicting unit (PU) level and transforming unit (TU) level. However, these approaches need to traverse all CU sizes to obtain the optimal CUs; and this is the main reason for its limited performance, which requires further improvement. In HEVC, PU and TU partitions are used for determining whether the CU should be split or nonsplit. It can be inferred that CU encoding time will be significantly reduced when the encoder directly measures the CU coding tree tailor. Accordingly, mechanisms based on low complexity CU encoding by combining early determination with other coding parameters have been successfully applied. A couple of works have focused on speeding up CU coding tree computing through early termination mechanisms [13–17]. Cen [14] proposed a fast CU depth decision mechanism by utilizing spatial correlations to achieve CU depth range determination. Song [15] presented an early merge mode decision method to avoid exhaustive mode checks for CU derived from recursive quad-tree partitioning. Some prominent researches [18–19] should be aware that more low complexity schemes continuously focus on CU coding tree structure prediction. Guo [19] proposed a fast CU size selection algorithm based on hierarchical quad-tree correlations, and the size of the current CU can be determined according to the subtree distributions of adjacent CUs. In general, most of the current researches aim to reduce coding tree complexity by searching a specific tree depth instead of traversing all depths. In other words, current approaches only focus on reducing the leaf nodes of the coding tree by lessening depth; which means that the CU coding tree continuous to computed from its maximum CU size. However, with polytropic video content and coding parameters, a best CU coding tree structure may have a variable maximum CU size and be searched by a scalable depth. CU coding tree structures that are very complex contributes a little to coding performance but wastes amounts of encoding time. In general, using befitting CU coding tree structures is the key to reduce computational complexity, while maintaining good coding performance. In order to further improve CU coding tree prediction with a thorough tailor, a new low complexity CU coding tree mechanism was designed in our work. The proposed method is designed to conform to optimal CU partition according to quantization parameter (QP) and content change (CC).

## 2. HEVC Coding Tree Structure

**Fig 1:**
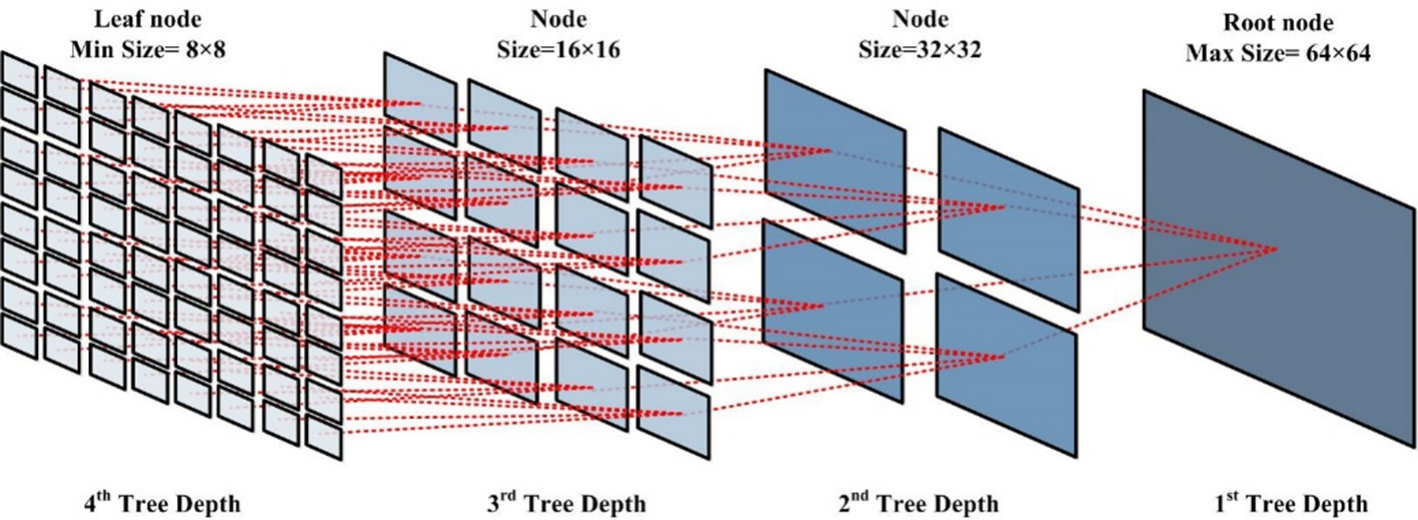
A complete CU coding tree in HEVC.

The hierarchical coding structure of HEVC is based on the coding tree structure of CU, as shown in Fig 1. Coding tree unit (CTU) is defined as a root node with a CU size of 64×64. The coding tree structure allows recursive splitting into four equally sized nodes, which starts from the CTU and stops when tree depth reaches the maximum. Maximum tree depth is defined as the maximum number of splits in the CU coding tree, and one split results from dividing a CU into partitions for prediction (to be described below). Thus, a CTU size of 64×64 and a maximum tree depth of four imply that the leaf node has a smallest CU size of 8×8. Therefore, coding tree partitioning allows a content adaptive coding tree structure comprised of CU64 (a block contained 64 × 64 pixels), CU32 (a block contained 32 × 32 pixels), CU16 (a block contained 16 × 16 pixels), CU08 (a block contained 8 × 8 pixels).

**Fig 2:**
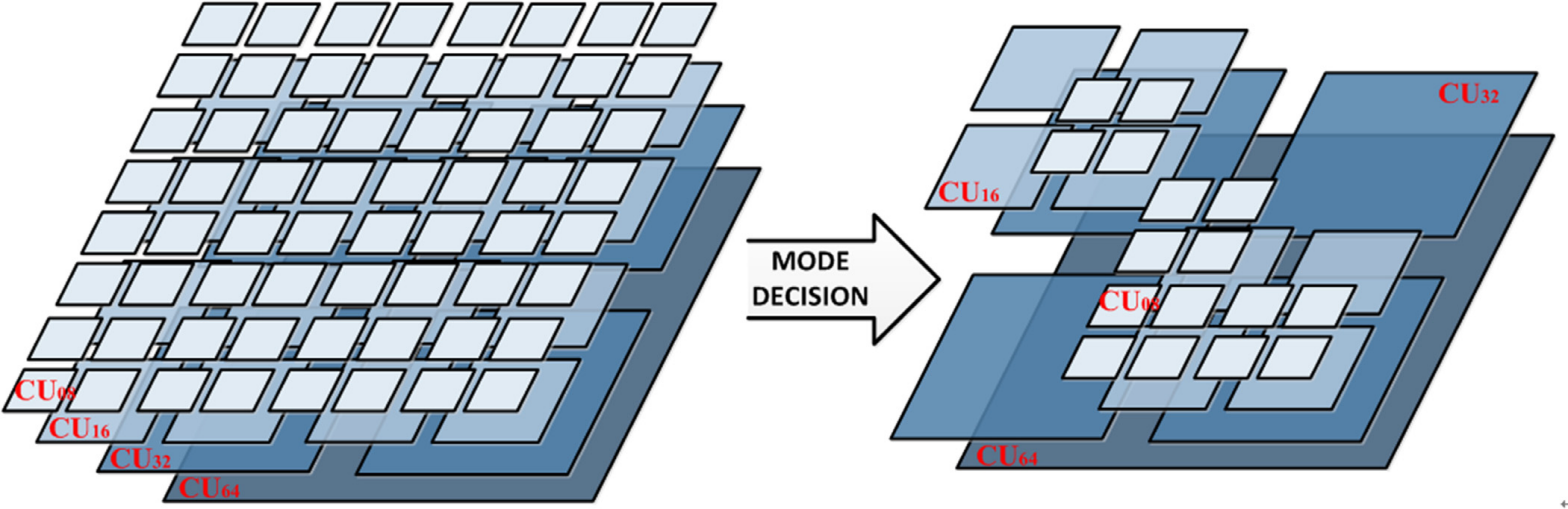
An example of CU mode decision for optimal CU size. CU64, CU32, CU16, CU08 represent the optimal CU size ranged from depth = 1 to depth = 4.

Each CU node is configured to use a particular prediction mode, which may be either intra prediction or inter prediction. Mode decision is a process that the encoder selects the optimal CU size for the current CTU by calculating coding costs *J*_mode_ of various CU modes. The cost function for mode decision *J*_mode_ is specified by

$$J_{\text{mode}}=\text{SSE}+\lambda_{\text{mode}}\cdot {\text{B}}_{\text{mode}}$$ (1)

where B_mode_, specifies the bit cost to be considered for mode decision, SSE represents the difference between two blocks with the same block size, and λ_mode_ is the Lambda value that is used for cost computation. For each CU, the following mode decision process is conducted in the HEVC encoder. The J_mode_ in each mode (MODE_INTER, MODE_SKIP, MODE_INTRA, PCM) is computed and J_mode_ is set to minimum CU coding cost. Then, a check is performed to determine whether the best coding mode is MODE_SKIP (early CU condition). The bit cost B_mode_ is updated by adding bits for the CU split flag and the minimum *J*_mode_ is recomputed. If the condition is true, do not proceed to the recursive mode decision at a smaller CU size. Otherwise, proceed to the recursive mode decision at a smaller CU size when the current CU depth is not at the maximum. This CU level mode decision is recursively performed for each CU depth. Final optimal CU size is determined by determining whether the current CU should split or not on the account of the minimum *J*_mode_ between *J*_mode_ of the current CU size and the sum *J*_mode_ of the four small sizes CUs. A CTU is divided into multiple CUs, as shown in Fig 2.

The flexible coding tree structure of HEVC contributes a significant improvement in coding gain. However, it causes a dramatic increase in encoding complexity, because the encoding process of HEVC needs to explore every single CU size from 64×64 to 8×8; in which the best PU and TU partition must be determined for all possible PU and TU sizes. This exhaustive mode checks results in an enormous increase in computational complexity.

## 3. Motivations and Analyses

The goal of reducing encoding complexity would be realized by tailoring a complete CU coding tree to avoid exhaustive CU size checks. This part would provide guidance on the aspect of the motivation for CU coding tree tailor and analyses of CU distribution, respectively.

### 3.1 Motivations for CU Coding Tree Tailor

**Fig 3:**
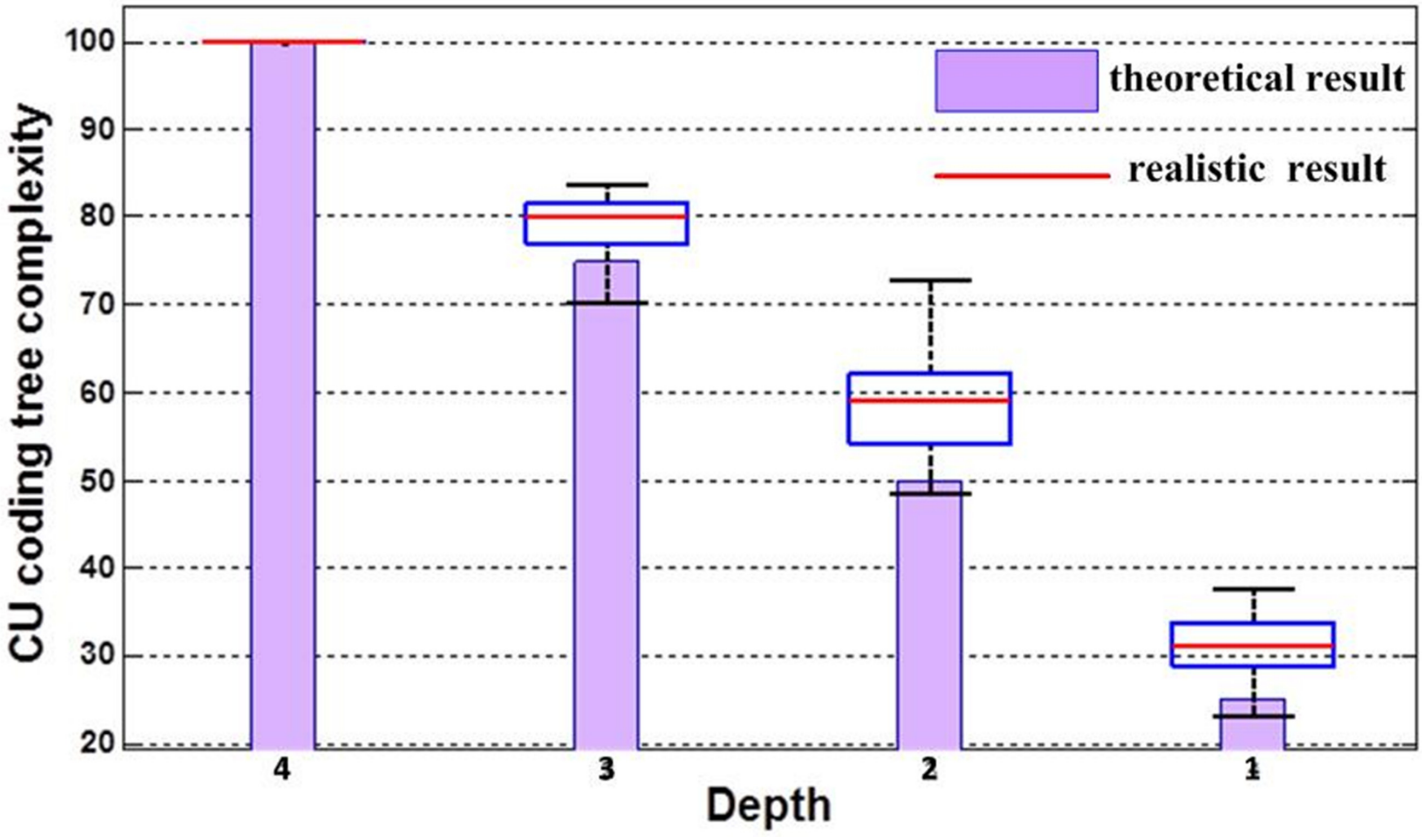
CU coding tree complexity.

The CU coding tree structure has an effect on encoding complexity due to the exhaustive traversal of its nodes for optimal CU size. A rough way to figure out the relationship between CU coding tree structure complexity and encoding saving is by performing a complete CU coding tree tailor. As stated above, a CU coding tree is made up of a root node and tree depth; thus, our experiment compares various tailored CU coding tree structures to the HEVC complete CU coding tree structure with 64×64 CTU and depth = 4. CU coding tree complexity is decided by both depth and CTU size. The small depth and CTU set, the lower CU coding tree complexity is. Fig 3 shows the CU coding tree complexity by varies depths and same CTU. It can be inferred that CU coding tree complexity proportionally drops with decreasing depth. The realistic result comes from the experimental statistic in Table 1, and the theoretical result indicates that the CU coding tree complexity drops 25% when depth drops 1. The realistic result of CU coding tree complexity reduction is close to theoretical results, but not as good as these results; because most theoretical results do not consider fast CU mode determination (early CU condition). Without loss of generality, CU coding tree complexity decreases when depth reduced.

**Table 1:** Encoding Time Saving (%) under Different CU Coding Tree Structure Compared with HEVC Coding Tree with Depth 4 and CTU Size 64×64.

Sequence	Depth = 3		Depth = 2			Depth = 1		
	CTU	CTU	CTU	CTU	CTU	CTU	CTU	CTU
	64×64	32×32	64×64	32×32	16×16	64×64	32×32	16×16
Traffic	19.62	20.72	51.39	39.59	45.81	73.48	71.27	64.96
Cactus	16.98	18.34	31.88	27.18	43.31	69.34	67.49	62.63
PartyScene	20.17	23.01	35.25	37.91	39.55	74.62	71.99	68.24
RaceHorses	23.59	29.74	32.26	38.14	44.45	76.90	70.84	66.98
Johnny	17.95	16.40	49.27	48.42	46.36	70.73	68.49	62.41
SlideShow	22.24	18.86	40.39	41.38	42.27	70.02	66.17	64.28
Average TS(%)	20.09	21.18	40.07	38.77	43.63	72.52	69.38	64.92

In addition to depth, CTU size is also a key to reduce CU coding tree complexity. As illustrated in Table 1, it can be observed that encoding time saving can be achieved up to 72.52% when the CU coding tree is a simple node and all CU sizes are 64×64; that is, the CU coding tree has a root node size of 64×64 and a depth of 1. Table 1 not only shows that a simpler CU coding tree structure can make a contribution in reducing encoding complexity, but also shows a trend that the larger the chosen CTU size and the more shallow the chosen depth are, the more time can be saved. However, attention should be given in an unavoidable problem, in which encoding accuracy severely drops when the CU coding tree structure is too simple. Thus, if the encoder contributes to saving encoding time while maintaining encoding accuracy, the CU coding tree tailor scheme should be built on a provision that whether some CU sizes with low probability can be tailored. In the next section, we will analyze factors affected by CU distribution.

### 3.2 Analyses of CU Distribution

**Fig 4:**
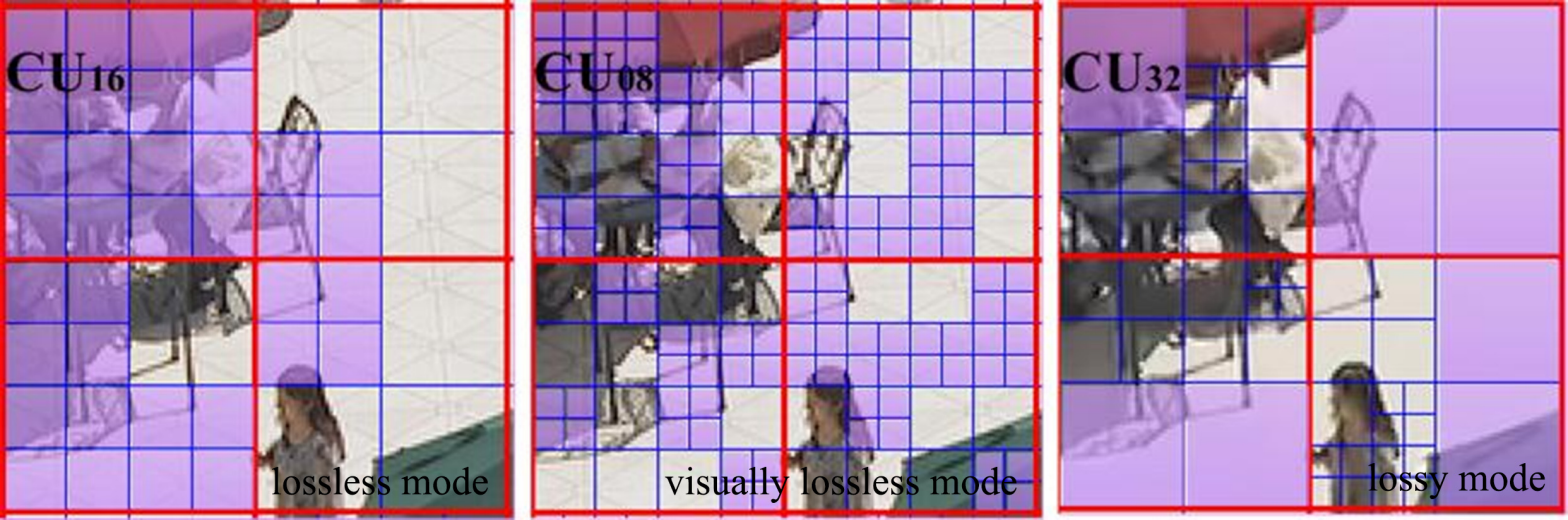
Optimal CU size after coding tree complete traversal for lossless mode with 63% CU size of 16×16, visually lossless mode with 59% CU size of 8×8 and lossy mode with 42% CU size of 32×32.

In order to investigate general CU distribution law, statistics are obtained under the widest QP range, from 0 to 47. Fig 4 shows the CU distribution probability with the encoded BQSquare sequence. It is shown that there is a high correlation between QP and the optimal CU distribution. It can be observed that CU sizes in a specific range occupy most percentages of the CU distribution in the same sequence. In general, the small QPs are highly likely to be encoded by the CU of small sizes. On the contrary, the large QPs are highly likely to be encoded by the CU of bigger sizes. In particular, the encoding condition is divided into three modes: lossless mode, visually lossless mode and lossy mode by different QP ranges. For lossless and visually lossless modes, a CU size of 64×64 has a quiet low probability; while a small CU size has a high probability, in which a CU size of 16×16 and 8×8 occupy 92.70% of the CU distribution under QP = 12. For lossy mode, the CU distribution changes dynamically, especially from QP = 22 to QP = 42. In this period of QPs, the probability of CU size 64×64 and 32×32 rapidly increase, while the probability of a CU size 16×16 and 8×8 rapidly declines; in which the sum of the CU size 64×64, 32×32 and 16×16 occupy 98.90% of the CU distribution under QP = 42. Therefore, it can be inferred that the percentage of CU distribution can be estimated under specific QP.

The analyses that CU distribution can be estimated are used to accelerate the CU encoding process. In detail, it is unnecessary to calculate all CU sizes from CU64 to CU08 in determining the optimal CU size. Encoders can only traverse a specific CU size range that occupies the most percentages of CU distribution probability. Other CU sizes outside of the specific range can be skipped, because these CU sizes are rarely used in the same sequence.

**Fig 5:**
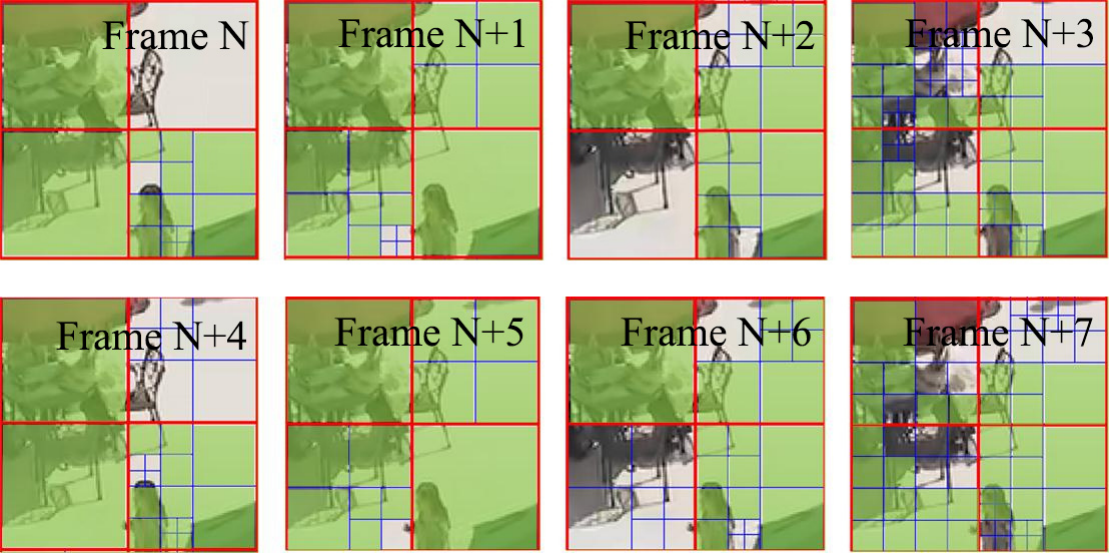
CU distribution caused by QP offset. The N^th^ frame and the (N + 4)^th^ frame have the similar CU distribution.

Fig 4 concludes that various QPs have a serious impact on CU distribution. However, the HEVC encoder has a more complex QP setting mechanism. HEVC inter coding is performed with different QPs according to the temporal layer. For different coding configurations, the QP of each inter coded picture is derived by adding an offset to the QP of the intra coded picture (QPI) depending on the temporal layer. For example, if a base QP is set to the QPI for first layer, the QP of the second layer QPL2 = QPI + 2, the QP of the third layer QPL3 = QPI + 3, and so on. This configuration leads to a nonuniform *J*_mode_ for different pictures by changing λ_mode_ defined in Eq 1. The λ_mode_ is the Lambda value used for cost computation by the equation:

$$\lambda_{\text{mode}}=\alpha \cdot W_{k}\cdot 2^{\frac{\left(QP-12\right)}{3}}$$ (2)

where *W*_*k*_, represents the weighting factor dependent to the QP offset hierarchy level of the current picture within a group of picture (GOP). This means that although a base QP has been set before encoding, each picture has its own QP; which depends on QP offset and results in a different *J*_mode_ for coded pictures, not to mention the different CU distributions for coded pictures. Due to this reason, it is important to investigate the CU distribution law of each frame based on GOP structure. Table 2 illustrates the QP and QP offset in low delay and random access for experiment. Table 3 and Table 4 show the CU distribution probability within different GOPSizes under QP = 32. It can be observed that there is a relationship between CU distribution and GOP structure. Fig 5 shows the CU distribution probability of BQSquare sequence. A CU size of 32×32 has the most probability in the first three pictures of a GOP. However, for the fourth picture, the probability of a CU size of 32×32 severely drops, while other CU sizes simultaneously increases markedly. It is noteworthy to mention that almost all GOPs follow the above rule. The reason is that different pictures have a same fixed QP offset in different GOPs, and the picture order count (POC) is also fixed in different GOPs. Hence, there is a similar CU distribution in adjacent GOPs, because the same POC has the same QP offset. Therefore, our first motivation for low complexity CU coding tree mechanism is that the ***N***^***th***^ frame and the (***N*** + **GOP size**)^***th***^ frame have similar CU distribution.

**Table 2:** Lossy Encoder Configuration.

**Low Delay**	
QP	22, 27, 32, 37
GOP Size	4
POC	1, 2, 3, 4
QP offset	3, 2, 3, 1
**Random Access**	
QP	22, 27, 32, 37
GOP Size	8
POC	8, 4, 2, 1, 3, 6, 5, 7
QP offset	1, 2, 3, 4, 4, 3, 4, 4

**Table 3:** CU distribution probability (%) with various videos and GOPSizes (Traffic: 2160×1600).

Frame	CU type	GOPSize = 4				Frame	GOPSize = 8							
**33**	**CU64**	76.79	71.43	66.07	16.07	**33**	28.20	64.60	68.50	83.00	81.70	68.70	85.50	83.10
**34**	**CU32**	21.43	22.32	23.21	44.64	**34**	34.45	22.80	21.10	13.65	14.75	21.25	10.90	13.20
**35**	**CU16**	1.79	4.46	7.13	26.79	**35**	27.95	10.55	8.90	3.02	3.15	8.22	3.23	3.23
**36**	**CU08**	0.00	1.79	3.58	12.50	**36**	9.40	2.05	1.50	0.33	0.40	1.83	0.38	0.47
**37**	**CU64**	75.00	73.21	69.64	16.07	**37**	27.80	62.90	71.00	83.10	82.60	68.30	85.10	85.60
**38**	**CU32**	20.54	17.86	27.68	42.86	**38**	36.50	24.35	20.95	14.05	13.75	22.85	12.25	12.05
**39**	**CU16**	4.46	6.70	2.68	21.88	**39**	26.57	9.88	6.73	2.33	2.97	7.32	2.33	2.23
**40**	**CU08**	0.00	2.23	0.00	19.20	**40**	9.13	2.88	1.33	0.53	0.68	1.52	0.33	0.13
**41**	**CU64**	75.00	71.43	73.21	17.86	**41**	26.20	61.10	69.20	83.00	82.40	68.40	81.00	79.90
**42**	**CU32**	21.43	22.32	20.54	42.86	**42**	37.85	24.55	21.90	13.75	14.00	21.50	14.75	15.85
**43**	**CU16**	3.13	4.91	5.36	17.41	**43**	26.88	12.03	7.38	2.93	3.33	8.20	3.70	3.60
**44**	**CU08**	0.45	1.34	0.89	21.88	**44**	9.07	2.33	1.52	0.33	0.27	1.90	0.55	0.65

**Table 4:** CU distribution probability (%) with various videos and GOPSizes (BQSquare: 416×240).

Frame	CU type	GOPSize = 4				Frame	GOPSize = 8							
**33**	**CU64**	35.71	28.57	10.71	3.57	**33**	0.00	25.00	35.71	57.14	57.14	28.57	60.71	60.71
**34**	**CU32**	42.86	49.11	62.50	26.79	**34**	16.08	50.00	35.71	23.21	28.57	42.86	25.00	25.00
**35**	**CU16**	18.97	17.63	24.11	52.01	**35**	45.21	20.54	23.21	19.64	14.29	22.32	14.29	13.39
**36**	**CU08**	2.46	4.69	2.68	17.63	**36**	38.71	4.46	5.36	0.00	0.00	6.25	0.00	0.89
**37**	**CU64**	39.29	17.86	17.86	3.57	**37**	0.00	17.86	32.14	53.57	53.57	28.57	53.57	46.43
**38**	**CU32**	41.07	57.14	54.46	25.00	**38**	16.07	44.64	39.29	28.57	28.57	44.64	32.14	39.29
**39**	**CU16**	17.63	19.64	25.22	47.32	**39**	48.21	28.57	21.43	17.86	16.96	21.43	12.50	12.50
**40**	**CU08**	1.34	5.36	2.46	24.11	**40**	35.71	8.93	7.14	0.00	0.89	5.36	1.79	1.79
**41**	**CU64**	32.14	10.71	7.14	0.00	**41**	0.00	21.43	28.57	53.57	50.00	39.29	42.86	57.14
**42**	**CU32**	45.54	65.18	67.86	27.68	**42**	14.29	48.21	46.43	28.57	32.14	37.50	41.07	30.36
**43**	**CU16**	19.87	20.31	22.32	50.22	**43**	51.79	21.43	17.86	16.96	16.07	19.64	13.39	12.50
**44**	**CU08**	2.46	3.79	2.68	22.10	**44**	33.93	8.93	7.14	0.89	1.79	3.57	2.68	0.00

**Fig 6:**
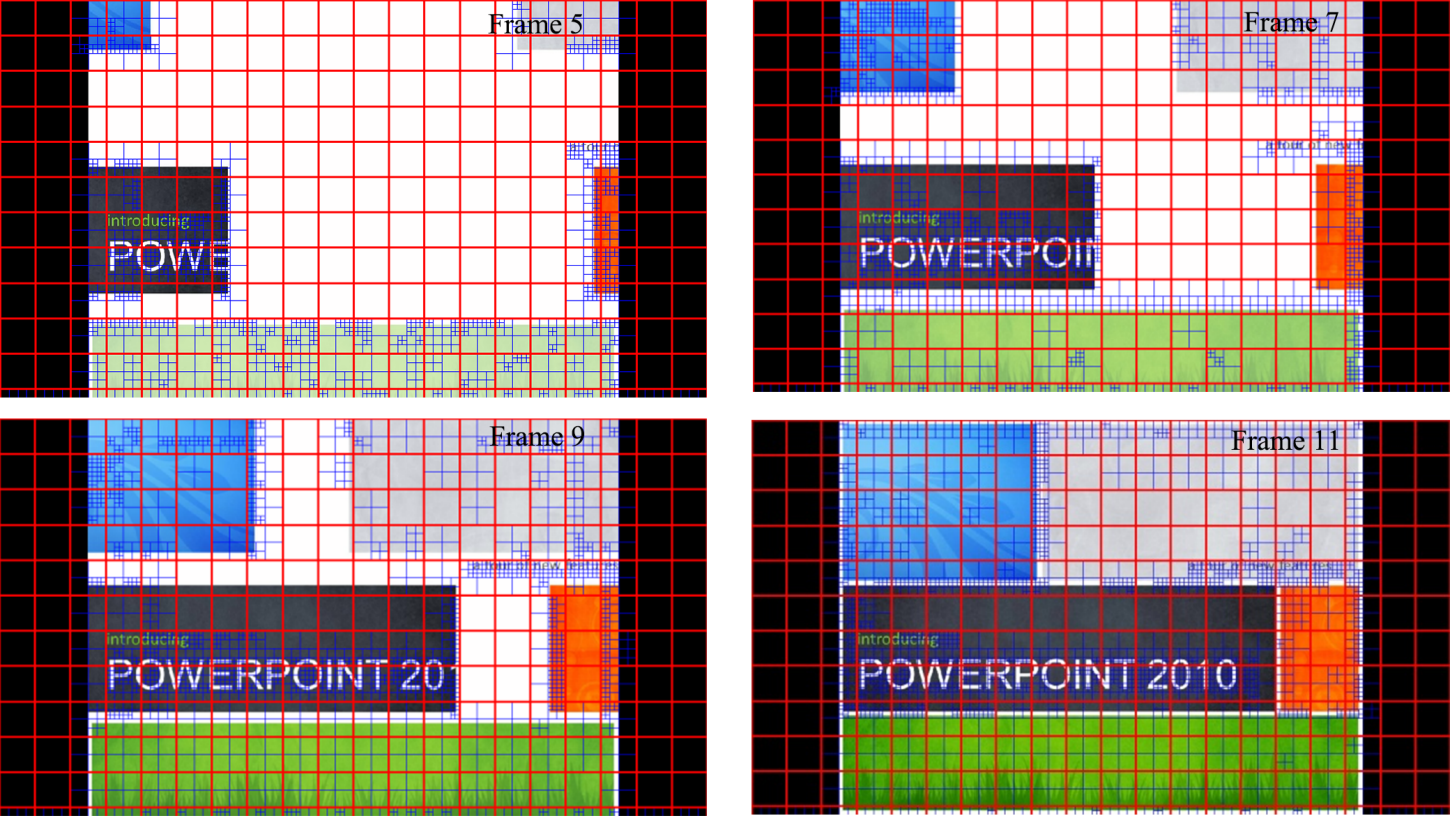
CU distribution probability caused by CC.

Another situation that would stir up CU distribution is CC. The CC of a nature sequence usually introduces new objects that make the current picture and future picture different from the previous picture. In other words, the steady CU distribution rule related to QP is broken. Due to this reason, statistic for CC is obtained. Fig 6 shows the CU distribution probability of an encoded SlideShow sequence without QP offset. These 12 frames represent a typical CC situation. From the **5**^***th***^ frame to the **11**^***th***^ frame, content dynamically changes by adding numerous texts and color blocks. When focusing on CU size 64×64 and 8×8, the probability of CU size 64×64 drops from 63.75% to 22.50% and the probability of CU size 8×8 increases from 16.59% to 56.28%. Therefore, excluding the effect of QP changes, CU distribution changed due to CC. Thus, our second motivation for low complexity CU coding tree mechanism is, CU distribution needs to be updated, otherwise it may be distorted due to CC.

## 4. Proposed Low Complexity CU Coding Tree Mechanism

The first motivation discovers that the *N*^*th*^ frame and the (*N* + GOP size)^*th*^ frame have a similar CU distribution, which means that there is explicit CU distribution redundancy in adjacent GOPs. The proposed method tries to utilize this CU distribution redundancy to achieve the optimal CU coding tree tailor by establishing a probabilistic model that computes the CU distribution redundancy in a GOP. Hence the key technique is proposed as using the *N*^*th*^ CU coding tree to predict the (*N* + GOP size)^*th*^ CU coding tree. However, the probabilistic model is not invariable due to CC, according to the second motivation. The necessity of a probabilistic model update in the process of video coding is not only to maintain the accuracy of the probabilistic model, but also to avoid error propagation to the later GOPs. Based on this, the proposed low complexity cu coding tree mechanism studies how a probabilistic model is established and how a new CU coding tree is established by using the probabilistic model. Moreover the low complexity cu coding tree mechanism studies how a probabilistic model is recomputed and how often an update performed.

### 4.1 Establish CU coding tree Model

First, a probabilistic model is established by computing the CU distribution in a coded GOP. An important function F_esta._ is established for establishing CU coding tree model. F_esta._ (CU, Frame|GOP_esta._) represents the probability of each CU size for each frame in the coded GOP. F_esta._ is performed by:

$$F_{\mathrm{esta}.}(\text{CU},{\text{Frame}|\text{GOP}}_{\text{esta}.})={\text{P}}_{ij}=\left[\begin{array}{ccc} \text{P}\left({\text{CU}}_{1},{\text{Frame}}_{1}\right) & \cdots & \text{P}\left({\text{CU}}_{1},{\text{Frame}}_{n}\right)\\ \vdots & \ddots & \vdots \\ \text{P}\left({\text{CU}}_{m},{\text{Frame}}_{1}\right) & \cdots & \text{P}\left({\text{CU}}_{m},{\text{Frame}}_{n}\right) \end{array}\right]$$ (3)

where CU_*i*_ refers to all kinds of CU sizes from 1 to *m*. P_*ij*_ is a two-dimension matrix, in which each element is calculated as:

$$\text{P}\left({\text{CU}}_{i},{\text{Frame}}_{j}\right)=\text{P}\left({\text{CU}}_{i}|{\text{Frame}}_{j}\right)=\frac{{\text{CU}}_{i}}{\sum_{1}^{m}{\text{CU}}_{m}}$$ (4)

where P is equal to a conditional probability that the frequency of a certain CU size within all CB (a block contained 4×4 pixels) in a frame. Next, a sign function Φ is calculated for CU coding tree tailor as follow:

$$\Phi =\delta_{ij}=\{\begin{array}{c} 0,\;{\text{P}}_{ij}<\sigma \\ 1,\;{\text{P}}_{ij}\geq \sigma \end{array}$$ (5)

notice that δ_*ij*_ is a two-dimension matrix that has the same size with F_esta._ and its elements contain only {0,1}. *σ* is used for deciding the lower limit of P_*ij*_. Φ is an important decider obtained from coded GOP and used to predict the probabilities of frames in later GOPs.

Second, before the encoder uses a complete coding tree to obtain P(CU_*m*_, Frame_*n*_) in a predicted GOP by traversing all CU sizes, a probabilistic model is established in advance by using Φ, to determine whether the probabilities of some CU sizes are equal to zero or not. With a similar form to F_esta._, a function F_predict_ used for CTPM predicting is designed. F_pred._ is performed by:

$$F_{\mathrm{pred}.}\left(\text{CU},{\text{Frame}|\text{GOP}}_{\text{pred}.}\right)={\text{P}}_{ij}{}^{\prime }=\Phi \cdot \left[\begin{array}{ccc} {\text{P}}^{\prime }\left({\text{CU}}_{1},{\text{Frame}}_{1}\right) & \cdots & {\text{P}}^{\prime }\left({\text{CU}}_{1},{\text{Frame}}_{n}\right)\\ \vdots & \ddots & \vdots \\ {\text{P}}^{\prime }\left({\text{CU}}_{m},{\text{Frame}}_{1}\right) & \cdots & {\text{P}}^{\prime }\left({\text{CU}}_{m},{\text{Frame}}_{n}\right) \end{array}\right]$$ (6)

notice that P_*ij*_′ is composed of {0, P′}, which means that the probability is zero or non-zero in the predicting frames. Ultimately, the F_pred._ is used for establishing a tailored CU coding tree for each frame in the predicted GOP. In order to obtain the new CU coding tree, the size of the root node and depth must be provided. Therefore the new coding tree is calculated according to CU_*i*_ in F_pred._ for each frame by: size of root node is MaxSize(CU|Frame) and size of leaf node is MinSize(CU|Frame), depth is log_2_MaxSize(CU_*i*_|Frame_*j*_) − log_2_MinSize(CU_*i*_|Frame_*j*_) + 1.

The size of the new CU coding tree is less than or equal to the complete CU coding tree which means the encoder can obtain an approximate optimal CU partition result without searching all the CU sizes.

### 4.2 Update CU coding tree Model

In later GOPs, the encoder uses the recomputed ${\text{F}}_{\text{esta}.}^{n}$ instead of the previous ${\text{F}}_{\text{esta}.}^{n-1}$. However, the key to guaranteeing coding efficiency is how often perform CTPU. As we know that frequent updating will introduce unnecessary computational complexity, meanwhile infrequent updating perhaps makes CTPM inaccuracy. Our method proposes two rules to decide whether ${\text{F}}_{\text{esta}.}^{n}$ is going to update or not eventually.

First, for the purpose to balance coding efficiency, firstly CTPU is performed based on frame rate parameter of each sequence. In generally, interval of CC is usually longer than one second. The frame rate parameter decides the frame numbers per second, namely the numbers of GOP per second. Therefore the CTPU period is defined as

T=Frame Rate+(Frame Rate)Mod(GOP Size) (7)

where (A)Mod(B) is equal to A − (AdivB) × B.

Second, although the CTPU period fixes the frame to be updated, the ${\text{F}}_{\text{esta}.}^{n}$ should not replace ${\text{F}}_{\text{esta}.}^{n-1}$ in the case that ${\text{F}}_{\text{esta}.}^{n}$ and ${\text{F}}_{\text{esta}.}^{n-1}$ have the similar CU distribution probabilities. Then performing CTPU depends on the matrix rank state which is usually measured for matrix equivalence between ${\text{F}}_{\text{esta}.}^{n}$ and ${\text{F}}_{\text{esta}.}^{n-1}$. Hence the CTPU is further defined as

$$F_{\mathrm{esta}.}^{n}=\{\begin{array}{c} \;F_{\mathrm{esta}.}^{n-1},\;\text{Rank}(F_{\mathrm{esta}.}^{n})=\text{Rank}(F_{\mathrm{esta}.}^{n-1})\\ \alpha \cdot F_{\mathrm{esta}.}^{n-1}+\left(1-\alpha \right)\cdot F_{\mathrm{esta}.}^{n},\;\text{Rank}(F_{\mathrm{esta}.}^{n})\neq \text{Rank}(F_{\mathrm{esta}.}^{n-1}) \end{array},\;\alpha \in \left[0,1\right]$$ (8)

where Rank(⋅) represents matrix rank. *α* is an equilibrium factor used for modify the performance of CTPU.

So far, CTPM contributes to deciding which CU size is skipped before encoder traverses a complete CU coding tree. CTPU devotes to maintaining the predicted accuracy meanwhile restricting the increase of computational complexity as much as possible.

## 5. Performance Assessments

### 5.1 General Experimental Configuration

The performance of the proposed low complexity CU coding tree mechanism is demonstrated in this section. To validate the effectiveness of the proposed method, gain or loss is measured with respect to HEVC test model version 15.0. Coding efficiency is measured by various resolution sequences arranged from 2560×1600 to 416×240. All sequences were tested under the common test conditions of HEVC standardization [20]. Other configurations are set as CTU varies from 64×64 to 16×16, partition depth varies from 4 to 1. For the experiments, the default fast encoding tools in HM15.0 are set to be turned on as an anchor. This means that our proposed method is compared with the original HM15.0 encoder under its best speedup condition.

To fully evaluate the contribution of the proposed low complexity CU coding tree mechanism under different conditions, individual performances were measured according to different settings, as follows: (1) Experiment I, performance of the proposed method for lossy coding; and (2) Experiment II, performance of the proposed method for visually lossless coding and lossless coding. The Bjøntegaard delta peak signal-to noise ratio (BDPSNR) and the Bjøntegaard delta bit rate (BDBR) [21] were used to evaluate the performance of the proposed method with respect to HEVC. Encoding time saving is calculated according to the following equation:

$$\text{TS}(\%)=\frac{E\text{nc}.\text{time}(\text{Anchor})-E\text{nc}.\text{time}(\text{Prop}.)}{E\text{nc}.\text{time}(\text{Anchor})}\times 100$$ (9)

### 5.2 Coding Performance Assessment

Experiment I is designed to verify the performance of the proposed low complexity CU coding tree mechanism for lossy coding. Experiment I has two parts: low delay condition and random access condition. Low delay applies to real-time communication, which has low delay request; while random access applies to support playback, video stream splicing, and so on. Table 5 shows the results of Experimental I for the proposed low complexity CU coding tree mechanism. Compared to HM15.0 best speedup condition, the proposed method yields a 28.67% average reduction in the total encoding time with a 1.39% average BDBR gain or 0.05 dB BDPSNR loss under low delay conditions, and achieves a 25.34% reduction in encoding time with a 1.18% BDBR gain or 0.04 dB BDPSNR loss under random access conditions. Particularly, the most achievement for encoding time saving is acquired from Class E under low delay. For the test sequences of Class E, the proposed method greatly saves total encoding time with negligible amounts of BDPSNR loss. This is because Class E has very large still background regions that exhibit little motion. Even though Class E performs the most complex CU coding tree, the probability of CU size 16×16 and 8×8 is quiet low. Therefore, the frame using the new CU coding tree without CU size 16×16 and 8×8 can achieve great coding performance as the anchor and markedly save encoding time. The proposed method is able to achieve considerable efficiency promotion with precise CU coding tree prediction.

**Table 5:** Performance of Proposed Mechanism Compared with HM15.0 under Low Delay Condition and Random Access Condition.

Class	Proposed method under LD condition			Proposed method under RA condition		
	BDPSNR (dB)	BDBR (%)	TS (%)	BDPSNR (dB)	BDBR (%)	TS (%)
Class A	-	-	-	-0.02	0.98	26.89
Class B	-0.03	1.18	28.35	-0.03	0.82	24.79
Class C	-0.10	2.14	29.01	-0.07	1.83	30.22
Class D	-0.06	1.32	23.47	-0.04	1.08	19.46
Class E	-0.01	0.93	33.84	-	-	-
Average	-0.05	1.39	28.67	-0.04	1.18	25.34

Experiment II is designed to verify the performance of the proposed method for lossless coding and visually lossless coding. Lossless and visually lossless compression is desired in many professional applications like medical imaging, surveillance systems, archiving systems, and so on. The specific configuration is shown in Table 6.

**Table 6:** Visually Lossless Encoder and Lossless Encoder Configurations under Low Delay Condition.

**Visually lossless Encoder Configuration**	
QP	0, 4, 8, 12
Lossless Mode	Disable
**Lossless Encoder Configuration**	
QP	0
Lossless Mode	Enable

Table 7 shows the results of Experiment II for the proposed low complexity CU coding tree mechanism. Compared to HM15.0 best speedup condition, the proposed method maintains a good quality reconstructed picture; and PSNR falls by barely 0.02 dB, which follows-up with a visually lossless image request. Bit rate increases by mere 0.82% and 0.46% for visually lossless coding and lossless coding, respectively, which keeps the HEVC high compression advantage. Most important of all, the proposed method achieves reducing encoding time up to 39.48% and 43.48% for visually lossless coding and lossless coding, respectively. On top of that, it can be seen that the proposed method is able to achieve better encoding complexity reduction to (visually) lossless coding than to lossy coding. The reason is the optimal CU distribution is either close to 100% or zero under low QPs, which makes the new coding tree simpler. Therefore, the proposed method gives a great approach for high quality image coding or video coding via low complexity.

**Table 7:** Performance of Proposed Method Compared with HM15.0 for Visually lossless Coding and lossless Coding.

Class	Proposed method for visually lossless coding			Proposed method for lossless coding	
	BDPSNR (dB)	BDBR (%)	TS (%)	Bit-rate Increase (%)	TS (%)
Class B	-0.01	0.33	49.35	0.25	49.19
Class C	-0.03	1.15	36.87	0.56	38.99
Class D	-0.02	0.85	23.07	0.48	30.37
Class E	-0.01	0.49	44.62	0.15	48.24
Class F	-0.04	1.29	47.50	0.84	50.61
Average	-0.02	0.82	40.28	0.46	43.48

The efficiency of optimal CU partitioning is described as the probability of obtaining the optimal CUs by performing a CU coding tree node once. For the anchor, the CU partition time is a constant determined by the number of complete CU coding tree nodes. The optimal CU must be one of the CU sizes of CU64, CU32, CU16 and CU08, and then the efficiency of the anchor is equal to 0.25. For the proposed method, the number of CU coding tree nodes is less than or equal to the anchor. Therefore, the proposed method obtains the optimal CU by performing less CU coding tree nodes. The efficiency of the proposed method most time overtops 0.25. Sometimes the proposed method cannot obtain the optimal CU partitioning result due to omit some of low probably CU coding tree nodes. This deviation is reflected on the BDPSNR drop and BDBR increase. In our experiment, the BDPSNR drops less than 0.05dB and BDBR increases less than 1.39%. However the deviation affects the coding efficiency slightly, which we consider it as the trade off on coding complexity reduction.

The efficiency of the proposed method is better than the anchor in lossless coding and visually coding. The proposed method used a CU coding tree with a root node size of 32×32 and depth of 3 to achieve the similar optimal CU partition results, compared to the anchor. As a matter of fact, for most sequences, the proposed method rarely performs a CU size of 64×64 under low QPs in HM. Hence, CU partitioning results of the proposed method with a root node size of 32×32 and depth of 3 are very similar to the optimal CU partitioning results measured by a root node size of 64×64 and depth of 4. On the contrary, with high QPs, the proposed method chooses not to perform a CU size of 8×8 due to the large number of still and flat background blocks. Hence, the proposed method may loss partial details in lossy coding. For lossy coding, although the CU partitioning results do not contain a CU size of 8×8, the reconstructed picture has little difference compared to HEVC partitioning result. The reason is that high QP has a more severe impact on quality degeneration than CU partition without a CU size of 8×8. Generally speaking, the low complexity CU coding tree mechanism successfully reduces CU encoding complexity by accepting an adaptive CU coding tree structure for each frame, and avoids complex computing and large memory requests, which makes it convenient to generalize in pervasive applications.

## 6. Conclusions

In this paper, a novel low complexity CU coding tree mechanism is proposed for reducing HEVC encoding time. In order to deal with the high computational complexity caused by the complete traversal of the CU coding tree in HEVC, CU distribution is explored and a low complexity CU coding tree mechanism is proposed for optimizing the CU coding tree tailor. The best discovery is that CU distribution is related to QP and CC. Moreover, the proposed method is based on GOP to implement the prediction, which makes full use of CU distribution redundancy. All the experiment results show that for lossy coding, the proposed low complexity CU coding tree mechanism achieves a 27% average encoding time reduction. For lossless coding and visually lossless coding, it is possible for the proposed method to achieve a 42% encoding time reduction, while maintaining the high quality of the original picture. The proposed low complexity CU coding tree mechanism breaks through the original CU structure by avoiding low probability CU traversal and reducing unnecessary encoding time with the almost similar compression performance. Fundamentally the proposed method improves the performance of HEVC real-time encoding effectively and can be combined with other fast video coding techniques to accelerate encoding speed. In addition, the proposed method is willing to devote its applications for various conditions, in which computational resources are limited.
